# Supplementary material for: Axolotl mandible regeneration occurs through mechanical gap closure and a shared regenerative program with the limb
Source: Dis Model Mech. 2024 Sep 27;17(9):dmm050743. doi: 10.1242/dmm.050743 (PMC11449444; doi:10.1242/dmm.050743)
Supplement: Supplementary information [file dmm-17-050743-s1.pdf]

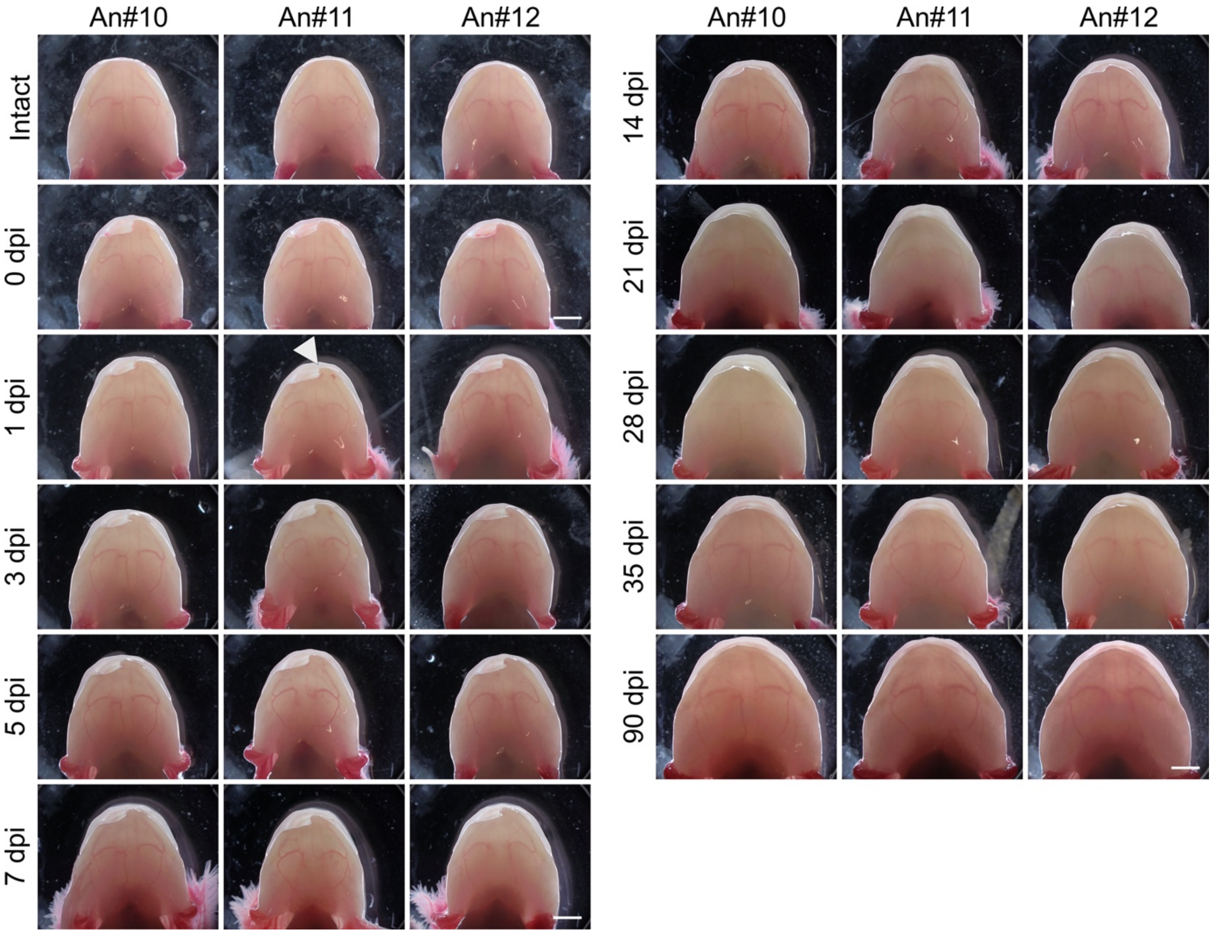

Fig. S1. Time course of lower jaw regeneration after full-thickness lateral resections in 3 representative individuals. Animals are pictured before (Intact) and immediately after resection (0 dpi), and at 1-, 3-, 5-, 7-, 14-, 21-, 28-, 35-, and 90 dpi. White arrowhead indicates medial edge of the defect. An#11 is used for time course in Fig.1. Scale bar: 5 mm.

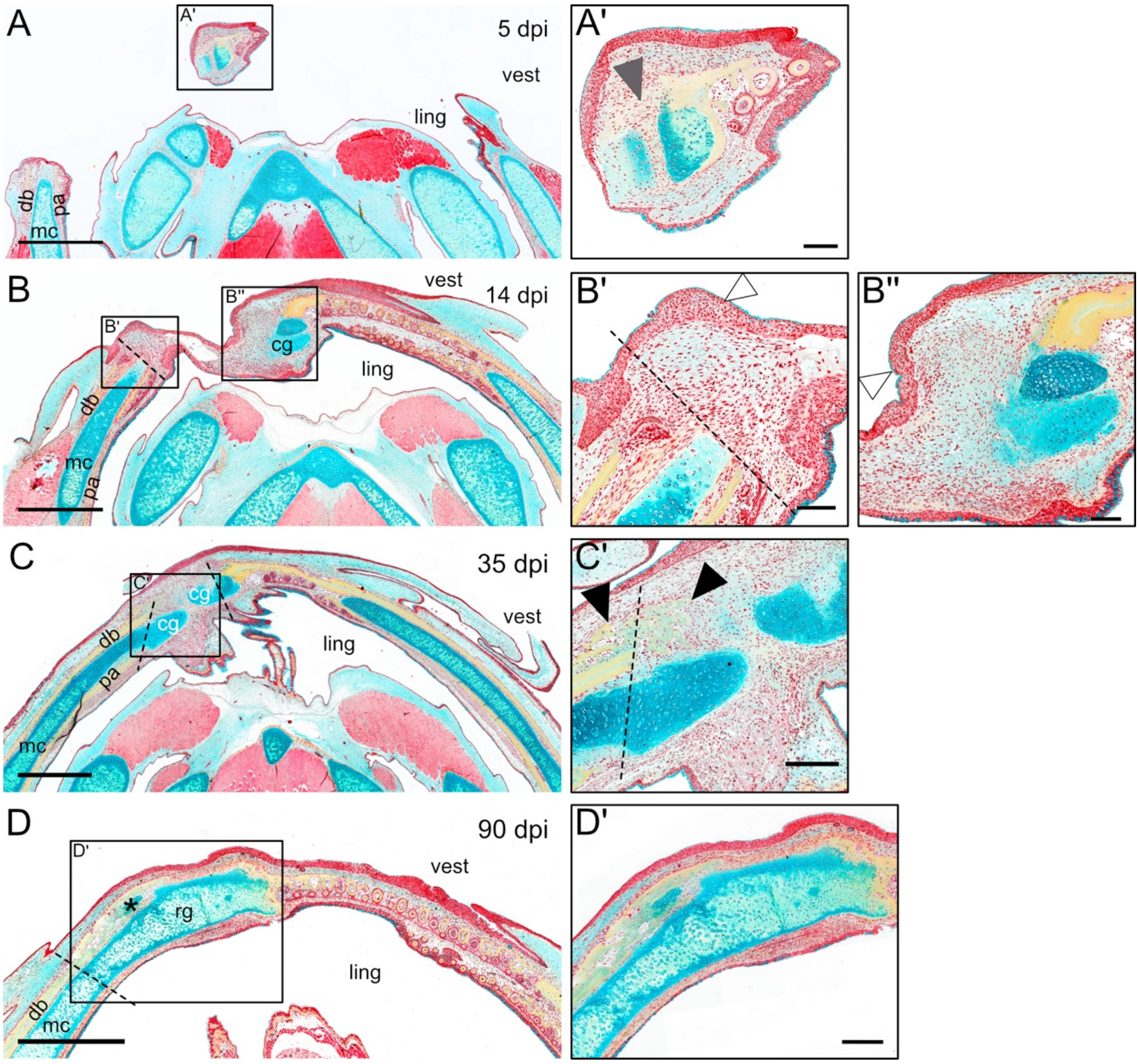

Fig. S2. Movat's pentachrome staining of longitudinal sections of regenerating lower jaws after full-thickness lateral resections at 5- (A), 14- (B), 35- (C), 90 dpi (D). Grey arrowhead in A' represents the medial symphysis. White arrowheads in B' and B'' indicate the wound epithelium. Black arrowheads in C' show intramembranous ossification. Asterisk in D indicates a pocket of cartilage. Dashed lines indicate the approximate site of resection. mc: Meckel's cartilage, pa: prearticular, db: dentary; vest: vestibular side; ling: lingual side; cg: cartilaginous growth; rc: regenerated cartilage. Scale bar in A-D: 2 mm, scale bar in A', B', B'', C': 250 µm. Scale bar in D': 500 µm. All of the sections correspond to different planes through the regenerated region in the same animals shown in Fig. 2. D corresponds to an intermediate section between Fig. 2E and F.

Disease Models & Mechanisms • Supplementary information

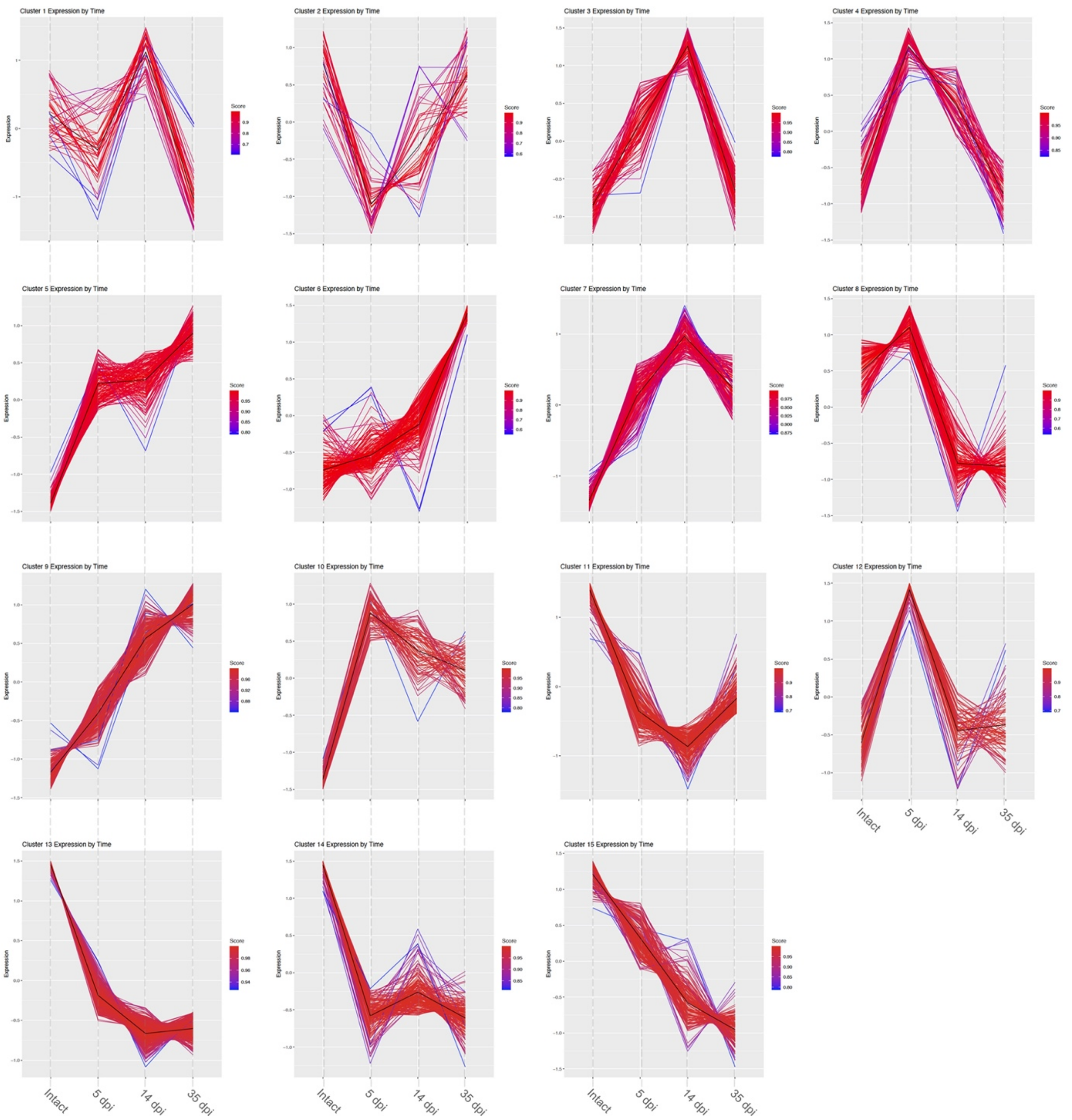

Fig. S3. 15 clusters capturing the gene expression of lower jaw regeneration in intact tissues and at 5-, 14-, and 35 dpi Color gradient indicates the Pearson Correlation of any given gene to the core of its corresponding cluster. Y-axis indicates expression as scaled z-scores.

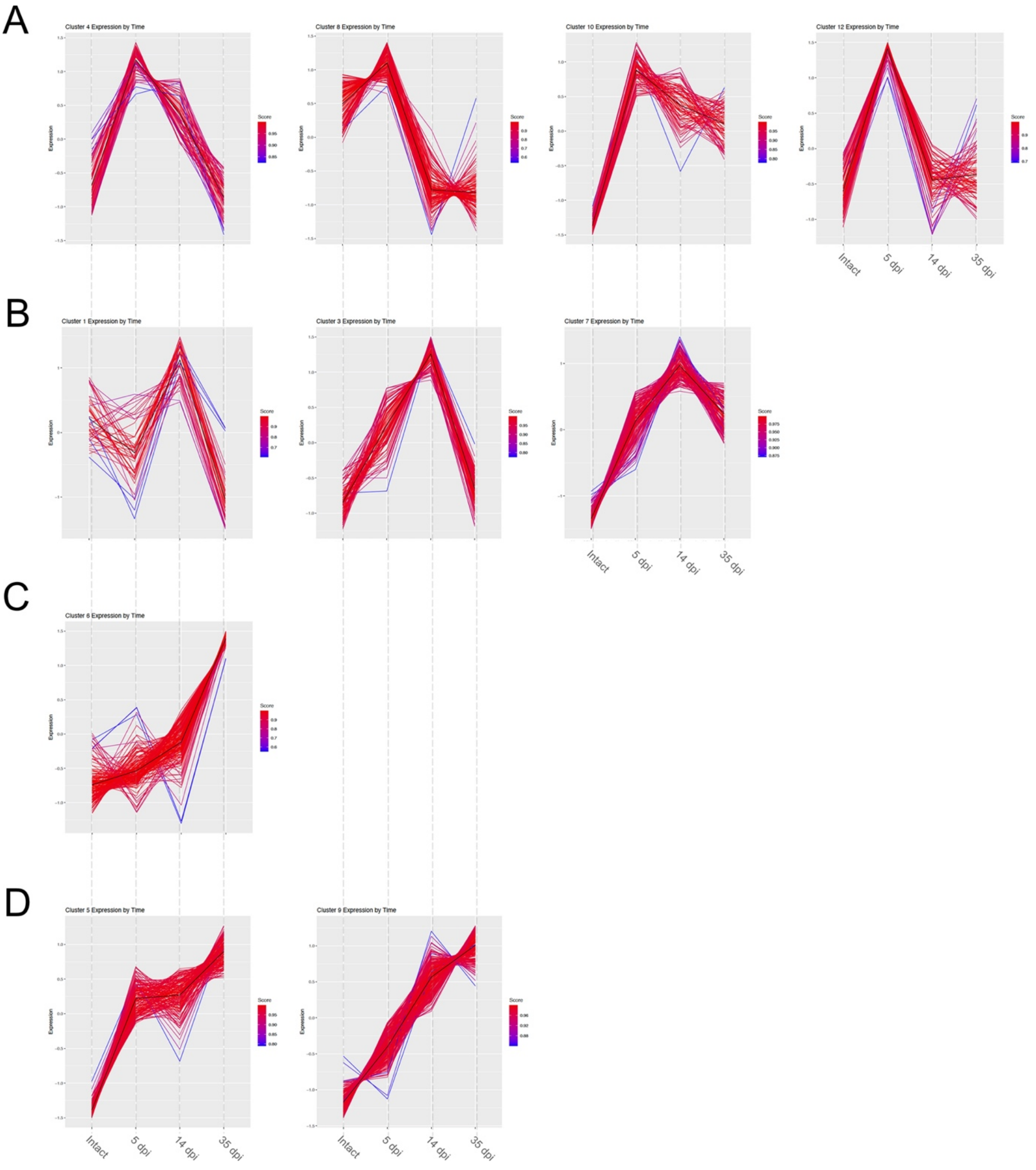

Fig. S4. Regenerating lower jaw cluster groupings for downstream analysis. A. Clusters 4, 8, 10 and 12 are grouped in the category *5 dpi Peak*. **B.** Clusters 1, 3, and 7 are grouped in the category *14 dpi Peak*. **C.** Cluster 9 is the only one composing the category *35 dpi Peak*. **D.** Clusters 6, and 9 are grouped in the category *General Rise*. Gradient indicates the Pearson Correlation of any given gene to the core of its corresponding cluster. Y-axis indicates expression as scaled z-scores.

Disease Models & Mechanisms • Supplementary information

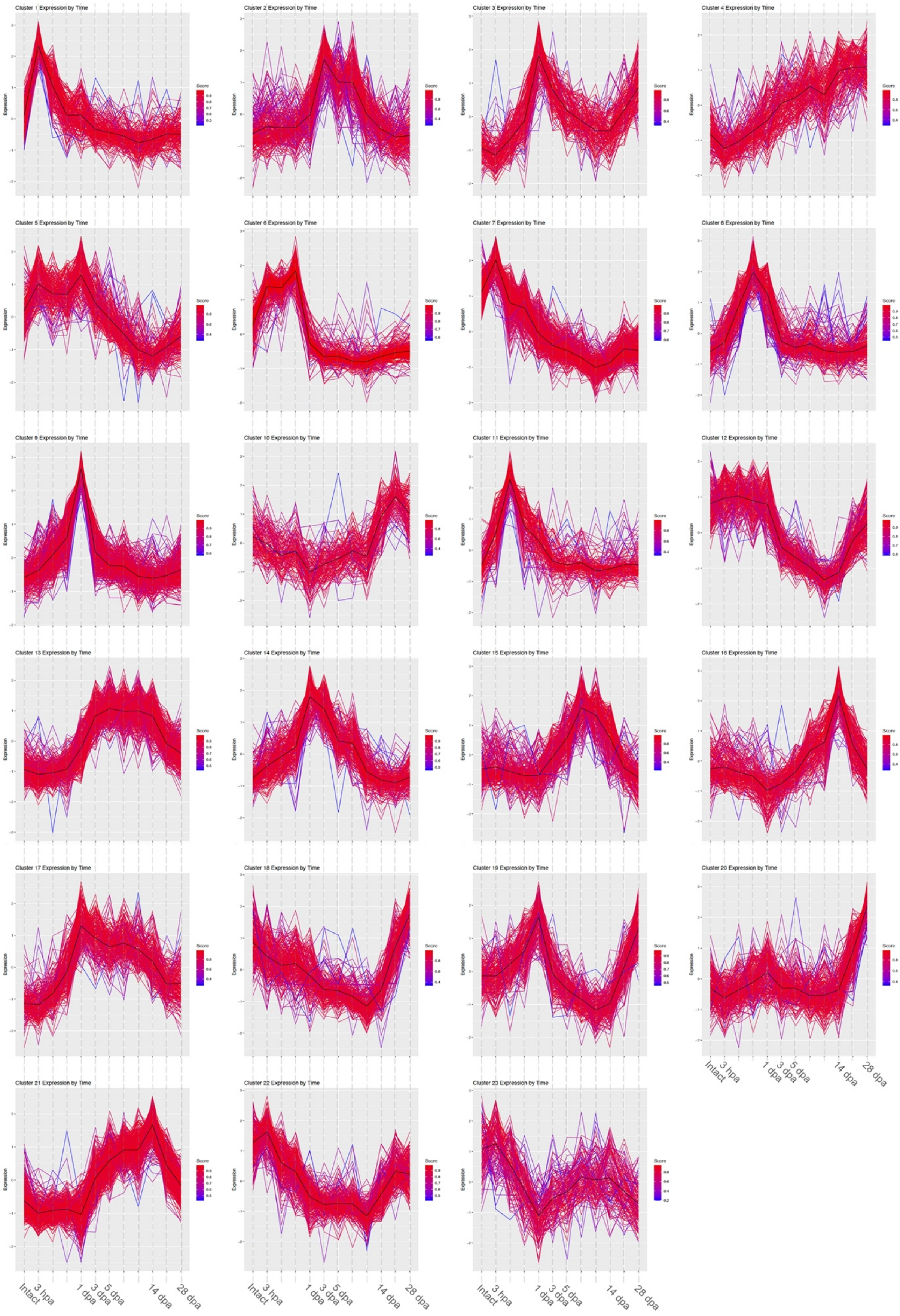

Fig. S5. 23 clusters capturing the gene expression of limb regeneration over time found by *k*-means clustering algorithm. Color gradient indicates the Pearson Correlation of any given gene to the core of its corresponding cluster. Y-axis indicates expression as scaled z-scores. Data from Stewart et al., 2013.

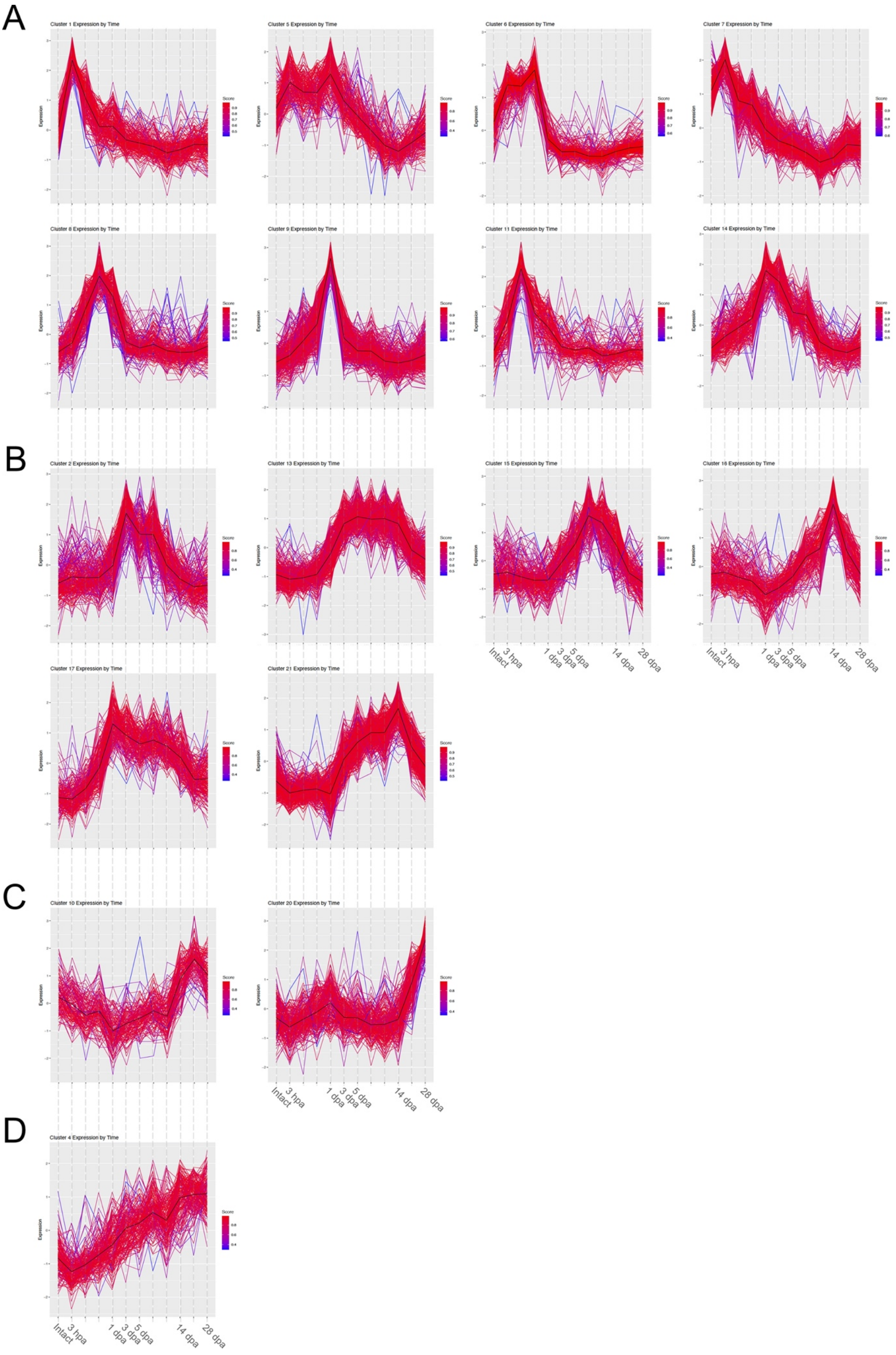
A
Cluster 1 Expression by Time
Cluster 5 Expression by Time
Cluster 6 Expression by Time
Cluster 7 Expression by Time
Cluster 8 Expression by Time
Cluster 9 Expression by Time
Cluster 11 Expression by Time
Cluster 14 Expression by Time
B
Cluster 2 Expression by Time
Cluster 13 Expression by Time
Cluster 15 Expression by Time
Cluster 16 Expression by Time
Cluster 17 Expression by Time
Cluster 21 Expression by Time
C
Cluster 10 Expression by Time
Cluster 20 Expression by Time
D
Cluster 4 Expression by Time
Expression
Score
Intact
3 hpa
1 dpa
3 dpa
5 dpa
14 dpa
28 dpa

Fig. S6. Regenerating limb cluster groupings for downstream analysis. A. Clusters 1, 5, 6, 7, 8, 9, 11 and 14 are grouped in the category *Early Peak*. **B.** Clusters 2, 13, 15, 16, 17 and 21 are grouped in the category *Mid Peak*. **C.** Clusters 10 and 20 are grouped in the category *Late Rise*. **D.** Clusters 6 is the only one in the category *General Rise*. Gradient indicates the Pearson Correlation of any given gene to the core of its corresponding cluster. Y-axis indicates expression as scaled z-scores.

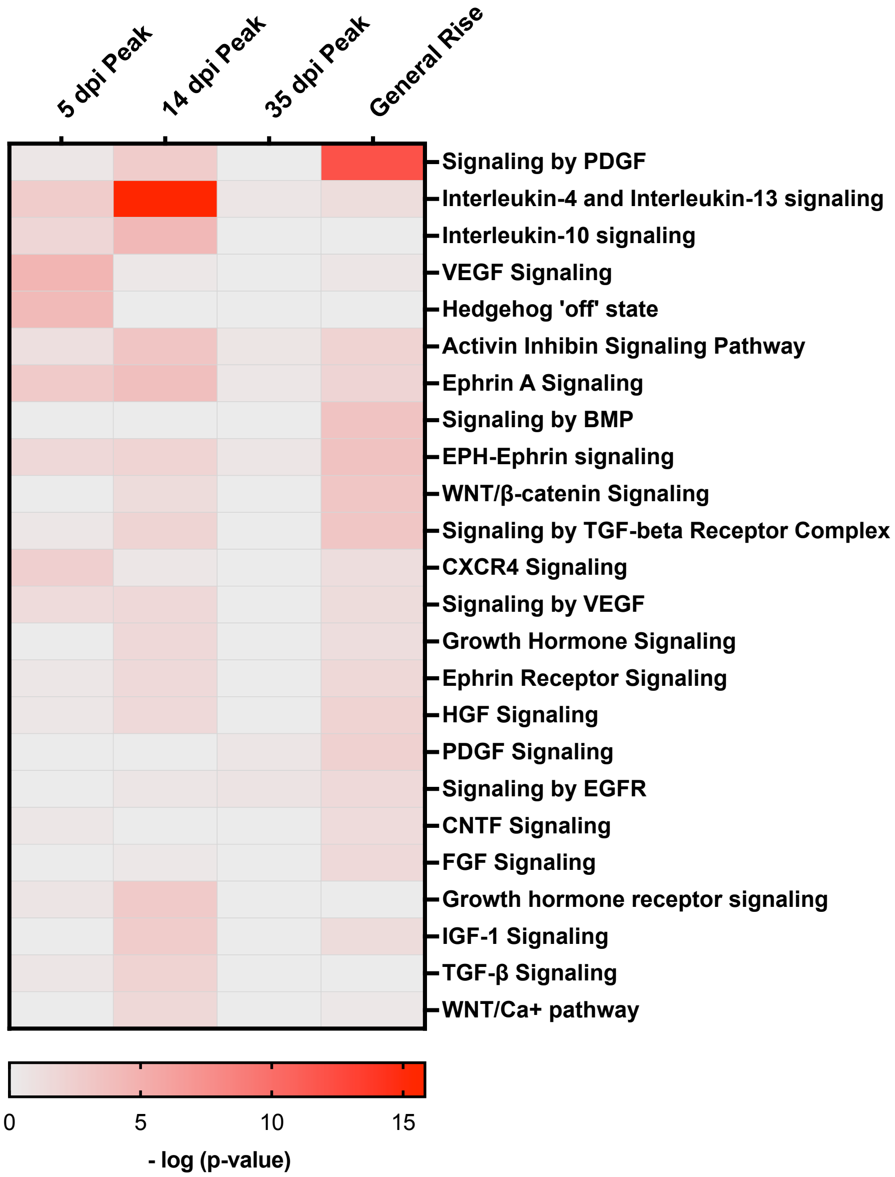

Fig. S7. Signaling pathway analysis in regenerating lower jaw groups. Scale bar represents the -log of the p-value. Higher values indicate a higher enrichment of a given pathway in each of the regenerating mandible groups.

Table S1. Size of intact mandibles, hemimandibles, and resection defects.

Available for download at https://journals.biologists.com/dmm/article-lookup/doi/10.1242/dmm.050743#supplementary-data

Table S2. Raw gene counts of mandible and limb.

Available for download at https://journals.biologists.com/dmm/article-lookup/doi/10.1242/dmm.050743#supplementary-data

Disease Models & Mechanisms • Supplementary information

Table S3. Normalized gene counts of mandible and limb.

Available for download at
https://journals.biologists.com/dmm/article-lookup/doi/10.1242/dmm.050743#supplementary-data

Table S4. Mean variance in gene expression (CPMs) of lower jaw and limb over time.

Available for download at
https://journals.biologists.com/dmm/article-lookup/doi/10.1242/dmm.050743#supplementary-data

Table S5. Cluster scoring of genes in lower jaw and limb.

Available for download at
https://journals.biologists.com/dmm/article-lookup/doi/10.1242/dmm.050743#supplementary-data

Table S6. Calculations of unique and shared genes in jaw and limb.

Available for download at
https://journals.biologists.com/dmm/article-lookup/doi/10.1242/dmm.050743#supplementary-data

Table S7. Gene ontology analysis of lower jaw *5 dpi Peak*.

Available for download at
https://journals.biologists.com/dmm/article-lookup/doi/10.1242/dmm.050743#supplementary-data

Table S8. Gene ontology analysis of lower jaw *14 dpi Peak*.

Available for download at
https://journals.biologists.com/dmm/article-lookup/doi/10.1242/dmm.050743#supplementary-data

Table S9. Gene ontology analysis of lower jaw *35 dpi Peak*.

Available for download at
https://journals.biologists.com/dmm/article-lookup/doi/10.1242/dmm.050743#supplementary-data

Table S10. Gene ontology analysis of lower jaw *General Rise*.

Available for download at
https://journals.biologists.com/dmm/article-lookup/doi/10.1242/dmm.050743#supplementary-data

Table S11. Gene ontology analysis of lower jaw *5 dpi Peak vs* limb *Early Peak*.

Available for download at
https://journals.biologists.com/dmm/article-lookup/doi/10.1242/dmm.050743#supplementary-data

Table S12. Gene ontology analysis of lower jaw *14 dpi Peak vs* limb *Early Peak*.

Available for download at
https://journals.biologists.com/dmm/article-lookup/doi/10.1242/dmm.050743#supplementary-data

Table S13. Gene ontology analysis of lower jaw *14 dpi Peak vs* limb *Mid Peak*.

Available for download at
https://journals.biologists.com/dmm/article-lookup/doi/10.1242/dmm.050743#supplementary-data

Table S14. Gene ontology analysis of lower jaw *14 General Rise vs* limb *Mid Peak*.

Available for download at
https://journals.biologists.com/dmm/article-lookup/doi/10.1242/dmm.050743#supplementary-data
